# A Statistical Model for Estimation of Fish Density Including Correlation in Size, Space, Time and between Species from Research Survey Data

**DOI:** 10.1371/journal.pone.0099151

**Published:** 2014-06-09

**Authors:** J. Rasmus Nielsen, Kasper Kristensen, Peter Lewy, Francois Bastardie

**Affiliations:** Technical University of Denmark, National Institute of Aquatic Resources (DTU AQUA), Charlottenlund, Denmark; Universitat Rovira i Virgili, Spain

## Abstract

Trawl survey data with high spatial and seasonal coverage were analysed using a variant of the Log Gaussian Cox Process (LGCP) statistical model to estimate unbiased relative fish densities. The model estimates correlations between observations according to time, space, and fish size and includes zero observations and over-dispersion. The model utilises the fact the correlation between numbers of fish caught increases when the distance in space and time between the fish decreases, and the correlation between size groups in a haul increases when the difference in size decreases. Here the model is extended in two ways. Instead of assuming a natural scale size correlation, the model is further developed to allow for a transformed length scale. Furthermore, in the present application, the spatial- and size-dependent correlation between species was included. For cod (*Gadus morhua*) and whiting (*Merlangius merlangus*), a common structured size correlation was fitted, and a separable structure between the time and space-size correlation was found for each species, whereas more complex structures were required to describe the correlation between species (and space-size). The within-species time correlation is strong, whereas the correlations between the species are weaker over time but strong within the year.

## Introduction

**Figure 1:**
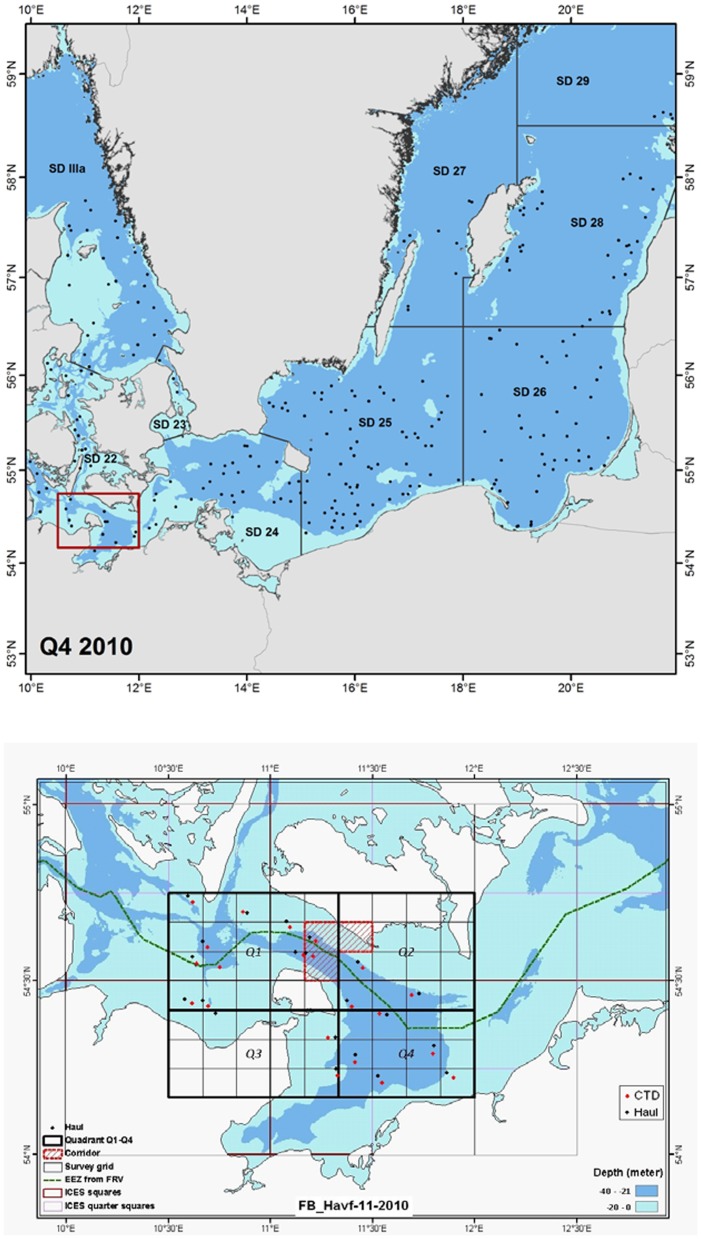
Investigation area and coverage of the stratified random and standardized ICES BITS trawl survey with new survey design according to Nielsen *et al.* [22] and Lewy *et al.* [23]. The stratified random haul locations are black dots (upper panel) and the additional coverage for the extended BITS survey in the Fehmarn Belt Area of the Western Baltic Sea with haul locations are indicated by black dots and associated hydrographical CTD stations as light dots (lower panel), exemplified for the quarter 4 2010.

A survey design was developed to extend the coverage of the standard ICES (International Council for Exploration of the Sea) Baltic International Trawl Survey (BITS; www.ices.dk) during 2009–2012 in the Western Baltic Sea (WBS). The aim was to enhance the power of the environmental impact assessment on the fish population dynamics of the establishment of the fixed transport link in the Fehmarn Belt area between Denmark and Germany in the WBS (Fig. 1). The resulting survey data with high spatial and seasonal coverage for a range of commercially important fish species are analysed with an extended variant of the Log Gaussian Cox Process (LGCP) statistical model [1], [2], [3].

Research survey data are often analysed under the assumption that the observations are independent, irrespective of trawl position, and distributed according to either extensions of the lognormal [4] or negative binomial distributions [5], [6]. Other studies have presented extensions of the multinomial distribution to account for data dispersion or correlation [7] or have used the geostatistical kriging approach to account for spatial correlations in the observations [8], [9]. Kristensen [1] and Lewy and Kristensen [2] estimated North Sea cod distribution patterns with the LGCP model using a statistical approach to determine spatial correlations between observations from surveys according to age. The overall formal structure of this model is given by Kristensen et al. [3]. The LGCP model is one of several models in the general family of parametric geostatistical methods, including hierarchical models and/or Gaussian latent variable models, that describe correlations in different dimensions including spatial correlation [8], [10]. An extension of the model was applied to mackerel (*Scombrus scombrus*) larvae survey data [11] based on additional temporal co-variance in spatial distributions.

In the present study, a similar extension of the LGCP model is applied to the standard and extended BITS survey data for Baltic cod and whiting. In contrast to most survey abundance models, which assume that the numbers by size caught in one haul are independent of numbers by size caught in all other hauls, the LGCP model utilises the fact that the correlation between numbers of fish caught increases when the distance in space and time between them in the sea decreases and, similarly, that the correlation between numbers caught of different sizes in a haul increases when the difference in size decreases. The model is further extended in two ways with the following aims. First, instead of assuming that the size correlation is used on the natural length-based scale, the correlation model is developed to allow the length scale to be transformed with, e.g., a logarithmic or a logistic function, with the aim of investigating whether this improves the correlation within and between species. The similarity between two individuals may indeed depend more on the ratio between animal sizes rather than size difference, suggesting, in this case, a log transformation of sizes. Second, the correlation with respect to time and space for different fish length groups between species is included in the model with the aim of analysing not only *intraspecific* size correlations but also potential *interspecific* correlation between species according to size. This is accomplished for fish species that are potential competitors or predators upon each other (e.g., [12], [13]) in the WBS such as cod and whiting.

The below hypotheses are accordingly tested:

**H01**: For each species (cod and whiting), the correlation in fish density depends on space and fish size. The correlation is a structured, separable size-space correlation. The space correlation depends on the geographical distance and the size correlation on the distance in transformed size between fish. The transformation is a specified function parameterised on, e.g., the natural, logarithmic, logistic or other transformation scales.
**H02**: For the two analysed species and for a given time period, there is a species correlation given the structured fish size and spatial correlations in fish density. Thus, there is a correlation in fish density between the two species.
**H03**: For the two analysed species, there is a time correlation between two time periods for a given species, given the structured fish size and spatial correlations in fish density. Thus, there is correlation in fish density between years for each fish species.

The purpose of the present study is to provide an extension of the methodology to obtain more precise estimates of relative fish density patterns, which are a prerequisite for environmental impact assessments, including spatial explicit fisheries and fish resource management and advice as well as marine management and spatial planning from a cross-sector perspective [14]. For the Baltic, a better estimation of underlying relative resource density and availability for fishery will, among other things, enable more precise description of fisheries and individual vessel-based specific fishing power, fish catchability, and partial fishing mortality [15], [16], [17], [18]. The methodology is extended here by not only using information on correlations in distribution patterns according to time and space between size groups within the different fish species [3] but also now considering the correlation in distribution of different sizes of groups between certain species that are expected to have interspecific interactions. The latter aspect can improve multi-species assessments and advice considerably by not only considering feeding analyses of fish in the multi-species models [19], [20] but also integrating information on actual mutual distribution patterns and their correlations of the species predating on or competing with each other in the marine ecosystems.

## Materials and Methods

### 1. Survey data used in the analyses

The international standardised ICES BITS survey is conducted in quarter 1 and quarter 4 of the year [21]. In 2001, the EU research project ISDBITS introduced a completely revised standard BITS survey [22], [23], [12], [21] with the aim of introducing new demersal survey gear and a revised stratified random survey design, expanding seasonal and geographical sampling to obtain better coverage of especially cod distribution areas in all life stages and also for other species, including herring (*Clupea harengus*) and sprat (*Sprattus sprattus*). In the traditional BITS, the participating nations used very different trawls, usually equipped with large bobbins, causing smaller cod to escape under the footrope [24]. ISDBITS employed new standardised survey trawls in addition to a standardised data sampling and processing design [22], [21]. The new sampling design has broader geographical coverage in the 1^st^ and 4^th^ quarters of the year (Fig. 1) and is based on random selection of haul positions. The number of hauls is selected partly according to the respective fraction in area of different depth zones in the Baltic ICES subareas (60% of the hauls) and the 5-year running means of cod aggregations (catch rates) (40% of the hauls) estimated in previous surveys). Furthermore, statistically robust and standardised inter-calibration methods to link old and new survey data time series have been implemented [23], [21]. Accordingly, the quality of the BITS survey data has, for the most recent 12-year time series, increased for demersal species, which allows obtaining of recruitment, density and abundance of age estimates at a higher coverage [12], [21].

Extended local-scale BITS surveying was conducted in the Fehmarn Belt area of the WBS from 2009–2012 on a quarterly basis using the same survey design (Fig. 1) and the data was linked to the standard large scale ICES BITS survey data time series. Figure 1 shows examples of coverage for the standard and extended BITS surveys. The extension has included extra trawl hauls for quarters covered by standard surveying (quarters 1 and 4) as well as repetition of the extension hauls here for the quarters not covered during standard surveying (quarters 2 and 3). Accordingly, data with higher spatial and seasonal resolution has been obtained to inform the statistical survey analyses with the LGCP model.

Several round fish, flatfish, and clupeoid species were abundant in the catches of the combined surveys. Initial analyses indicated that the species and size correlations do not have a simple structure but are rather variable for most of the species combinations. This variation was expected as the species-specific habitats and biological inter-specific relations are likely to be different according to size. However, consistent density patterns over years and quarters were found according to size-specific abundance distributions for cod and whiting. The detailed distribution patterns of cod and whiting according to size group are described in Supporting Information Appendix A for the period 2009–2012, quarters 1 and 4. The present study concentrates on model runs with cod and whiting data from 2009 and 2010, quarter 4. The raw data analysed here consists of the number of fish caught by 1-cm size class per haul.

### 2. Statistical model used and its further development

The LGCP model provides, similar to other models in the family of correlation models [8], [10], unbiased relative densities with a high resolution in time and space and by size/age for survey data by predicting and interpolating unobserved densities at any location in the covered area [1], [2], [3]. The formal model and its hierarchical structure are presented in Kristensen et al. [3] with a description how the model estimates latent, unobserved variables and how the goodness of fit (GOF) is determined (the latter is in the supplementary material). It is a counting model describing the discrete catch in number of observations, including zero observations. The model estimates spatial and temporal correlations between observations and includes zero observations, i.e., no-catch hauls, and over-dispersion parameters (Eq. 1) to enable analysis of all underlying survey data distributions. The LGCP model is a multivariate Poisson-lognormal distribution model, meaning that the catches in number observations are Poisson-distributed with mean densities following a multivariate lognormal distribution. The Poisson process is regarded as the sampling process generated by the fishing where there is an assumed spatial correlation between densities as a decreasing function of the geographical distance between them. The model parameters are obtained by maximum likelihood enabling interpolation and prediction of unobserved densities at any point in space and time and enabling goodness-of-fit tests [2], [3].

The models considered are characterised by explicit modelling correlation in space, size and species of survey density data. Single species, multispecies, and multiyear extension models are described.

### 3. The single species model, including size and space correlation

The correlation structure describes the spatial distribution of a single species of all size-classes *for a time-snapshot*. Here, a snapshot refers to approximately one month, i.e., the duration of the surveys analysed. The fish density modelling is based on considerations and testing of the processes acting on three different spatial scales (Hypothesis H01):

1. The large spatial scale size class variation is assumed to be an unstructured size distribution in the sense that the log-density of a size class *s* has a large scale mean of 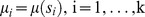 where 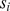 denotes size class *i* and *k* is the number of size classes.
2. Spatial variations in log-density, 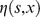, of a point in the space, 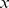, for a given size are assumed random by nature, with some structure due to fish behaviour and ecology, as fish of similar sizes are expected to occupy the same spatial areas.
3. Small spatial scale variations, 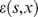, are assumed correlated across size-classes because of possible size-dependent schooling fish behaviour. Small scale variations can potentially be dominating in magnitude.

These three components suggest a model of the log density 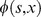 of a size-class 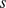 in a spatial point 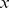 of the form 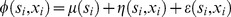
(Eq. 1)


It should be noted that 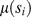 includes the combined effect of large scale size distribution in the sea and the selection of the catch process (including gear selection, duration of haul and other global effects of the catch process). On the log scale, 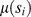 is the sum of these two effects. In context of the present study, 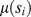 should be considered as a nuisance parameter because here, we are only interested in the size-space correlation, 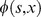, governing the log density as function of size and space. We make no assumptions on the structure and distribution of the combined large scale size distribution and gear selection, 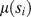, which accordingly is unconstrained. As a part of the model validation, the consistency has been checked between estimates of 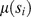 versus the spatial averaged count observations (CPUE, catch per unit of effort) across sizes, and the values were found to be consistent. The unconstrained model used here is in contrast to the approach in Kristensen et al. [3] where an *a priori* model for the 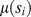 values is used based on the parameterised functions of gear selection and the decaying size spectrum. The present approach avoids such assumptions.

The process 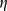 is defined through a covariance function. First, assuming separability between size and space, the covariance between two distinct size-classes at two different positions is 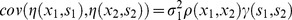
(Eq. 2)where 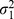 describes the magnitude of the process, and 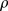 describes the spatial and 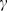 the size correlation.

In the same manner, the small-scale noise contribution 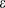 is defined through its covariance function 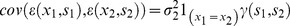
(Eq. 3)stating that this contribution only acts locally in space (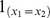) with the size correlation, 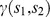, and with a total magnitude determined by 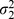. The size correlation, 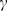, is assumed to be the same for the covariance of both 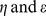. To understand the impact of Eq. 3, it is useful to view it in context of the stochastic processes in the following two scenarios. (1) For a fixed 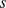, the 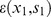
as function of 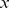 becomes white noise with intensity 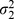. This reflects the uncertainty of the catch process when repeating a haul at a nearby position (we never have observations at exactly the same position with total spatial overlap). (2) For a fixed 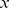, the 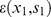
as function of 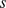 is correlated according to 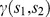. This reflects the within-haul size correlation. For further detailed reasoning and field ground evidence of this effect (Eq. 3), we refer to Kristensen et al. [3].

**Figure 2:**
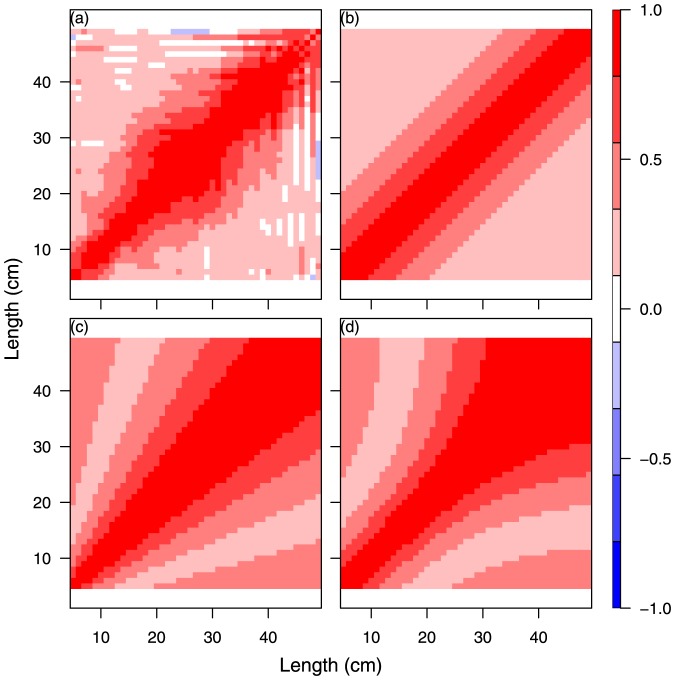
Comparison between fish size correlation matrix from different single-species model specifications for cod year 2009 quarter 4. SS1, Unconstrained free size correlation structure given as a positive definite correlation matrix (a); SS2, natural untransformed scale (b); SS3, log scaled (c); SS4, logistic scaled (d).

Next, we turn to the question how to parameterise the spatial correlation between two points 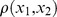 and the size correlation between two size groups 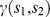. Most often, e.g., in kriging [25], the spatial correlation 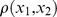 is assumed to be a function of the Euclidean distance 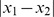. This, however, does not account for the possible complex geographical structure and variability of the sea. Rather, it is desirable to compute the covariance accounting for all possible paths to get from 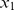 to 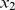 through the water area, with short paths weighing more than long paths. This feature is obtained by modelling 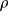 by using a Gaussian Markov random field [3], [26]. This means that, instead of modelling the covariance, the precision matrix *Q* is the basis for the modelling (*Q* is the inverse covariance matrix): 
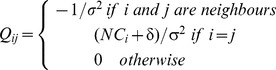
(Eq. 4)where *i* and *j* are grid points; 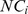 is the number of neighbours of the grid point 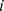, on a lattice grid (cell size 20*20 km); 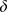 and 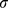 are positive parameters of the random fields. If point 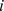 is an inner point, 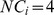 while boundary points have fewer neighbours. In Eq. (4), the spatial correlation increases when 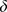 decreases, and the correlation between two points depends on the geometry of the grid. The properties of the Gaussian Markov random field co-variance (*Q^−1^*) generated from Eq. 4, which gives a decreasing correlation according to distance, taking into account the geometry of the grid, is shown in Figure S1. Another example of this is shown in Kristensen et al. [3 in Fig. 2D].

Regarding the size correlation 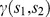, there are a number of options. The first option is the free unconstrained correlation 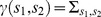 where the only requirement is that 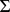 must be a positive definite correlation matrix. This model is called the *unconstrained size correlation model*.

It is convenient to reduce this model to a simpler structure where the correlation between size classes only depends on the distance between the size classes. However, this assumption has been modified to account for more complex correlation structures. Instead of just applying a correlation function, where the correlation decreases when the distance increases, we further extend the autocorrelation function between sizes with a periodic factor in Eq. (5): 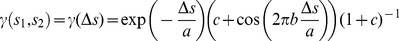
(Eq. 5)where the distance in size (cm) is 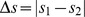. Note that this autocorrelation in one-dimension (size) is permissible (positive definite) according to Bochner's theorem [26]. The function contains the free parameters 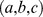, where 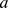 is a scale parameter; 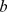 describes the periodicity; 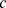 describes the minimal amplitude of the oscillations. Thus, the correlations can become negative. In the case of 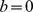, an exponential decreasing correlation is obtained. When 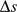 increases, the oscillation amplitude decreases towards zero, and the correlation converges to zero. The Eq. (5) size correlation model is flexible and allows for a possible decrease in correlation between close fish sizes (small distance in size) up to a certain level and then increases again for very different fish sizes (large distance in size), which may occur when smaller and larger fish occupy the same areas.

Third, instead of the distance between actual fish sizes, we may alternatively let the correlation depend on the distance between transformed sizes. For example, the similarity between two individuals may depend more on the ratio between animal sizes rather than size difference, suggesting, in this case, a log transformation of sizes. In addition to considering the distance on the natural scale, we thus as well consider the log and the logistic transformations such that the distance in the log case is defined as 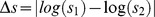. A logistic transformation is also investigated (Eq. 6) taking into account that the rate of change in distribution is small between the 1-cm groups for smaller and larger fish (high correlation), whereas for the medium-sized fish, the change in distribution is fast between 1-cm-groups (lower correlation). 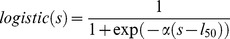
(Eq. 6)


The eq. (5) models, where 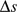 is based on the natural, the log scale and the logistic scales, are denoted models *parameterised on the natural, the log and on the logistic scale, respectively*. L50 is the size where we observe the highest rate of change in the spatial surface, and alpha measures that rate (the higher alpha is, the higher rate the rate is).

### 4. The multi-species extension of the model, including species correlation

The models from the previous section can be applied independently for two species 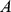 and 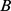: 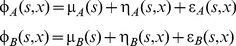
(Eq. 7)where the correlation patterns of the stochastic processes 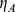, 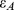, 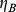 and 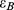 are estimated separately for each species.

In particular, the terms 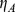 and 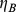 are independent and therefore have a covariance matrix of the form 
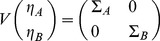
(Eq. 8)


In a multi-species context, the dependence between 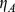 and 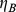 needs to be introduced. We describe and test two species correlation models: the *unconstrained species-size extension* and the *separable species-size extension*.

#### 4.1 The unconstrained species-size extension

Let 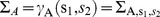, and 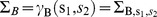 denote the size-correlation matrices of species A and B, respectively. The unconstrained extension of the correlation for the combined set of species A and B is then 
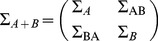
(Eq. 9)where 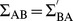 of dimension 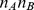 is free to choose with the only requirement that 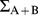 is positive definite. The Supporting Information Appendix B (part 3) shows that this requirement is fulfilled if 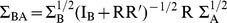, where *R* is any matrix of dimension 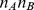 and where 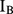 is the identity matrix.

The following properties hold for this extension:

- In terms of appropriate parameterisations, it has the right marginals for species A and species B, as selected from a prior single-species analysis.
- It has species independence as a special case (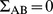), so that the independence assumption can be formally tested. Note, however, that this is generally a rather weak test for independence given the high degrees of freedom. Thus, it is desirable to reduce the model first to achieve a higher power of the independence test.

The unconstrained species-size extension of the model assumes that the random field parameters, 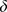 (Eq. 4), affecting the degree of spatial correlation for each of the species are identical, i.e., 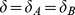. To conclude the construction of a space-size-species random field, the two terms 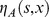 and 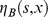 (Eq. 7) are tied together through the space-size-species covariance matrix 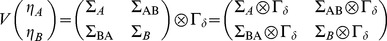
(Eq. 10)where 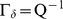 is the inverse of the precision matrix Q of the Gaussian Markov Random Field and where 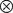 denotes the Kronecker product [26]. Eq. (10) states that *space* and the combination *(species, size)* are separable factors. The dimension of 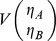, the quadratic covariance matrix, is 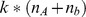, where *k* is the number of spatial gridpoints considered. As an example, the model states that the covariance between, e.g., 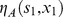 and 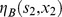 should be found as the product of the spatial covariance 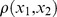 and the combined species-size correlation of the pair 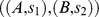. Note that separable extension (Eq. 10) of permissible covariances (e.g., one in size and one in space, i.e., multi-dimensional) is always again permissible according to the rules following the Kronecker product [26].

The multispecies model based on Eq. (10) with species-specific size correlation as defined by Eq. (2) is denoted the *unconstrained species-size correlation with species-specific size correlation*. This means that the parameters in Eq. (5), *a*, *b* and *c*, depend on the species. A sub-model with a common size correlation (i.e., *a*, *b*, and *c*, not depending on species) and denoted *unconstrained species-size correlation with common size correlation* is considered as well.

#### 4.2 The separable species-size extension

These models are based on and are sub-models of the unconstrained species-size correlation with common size correlation and attempt to measure the correlation, both within and between species, through the distance (dissimilarity) between transformed size groups.

More precisely, let *t* be a size-transformation function for the two species A and B. Consider two size groups 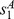 and 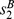 for the two species, and define the covariance between them as 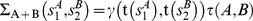
(Eq. 11)where 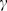 (Eq. 2) is a common correlation function valid for both species on the transformed size scale, and 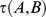 denotes the overall species correlation between A and B, i.e., 
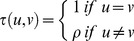
(Eq. 12)where u and v are in {u,v}
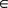
{A,B}. This MS3 model based on Eqs. (10), (11) and (12) is denoted the *separable* model and 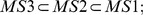 See Table 1.

**Table 1:** Overview of the models considered and tested, where single, multispecies and multiyear models are covered. In addition, the hierarchical structure of the model testing is indicated.

Type of Model	Model		Parameters
Single Species - including size and spatial correlation	SS1	Unconstrained	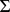 , 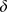 , 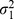 , 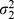
	SS2	Structured parameterised on natural scale	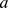 , 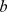 , 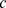 , 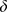 , 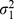 , 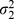
	SS3	Structured parameterised on log scale	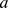 , 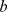 , 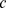 , 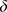 , 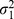 , 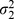
	SS4	Structured parameterised on logistic scale	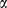 , 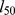 , 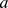 , 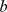 , 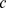 , 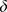 , 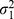 , 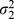
Multi-Species - including species, spatial and size correlation	MS1	Unconstrained species-size correlation with species specific size correlation	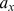 , 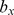 , 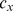 , 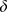 , 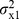 , 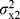 , *x = species A B*, 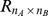
	MS2	Unconstrained species-size correlation with common size correlation	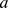 , 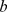 , 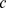 , 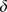 , 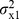 , 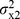 , *x = A B*, 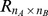
	MS3	Separable model	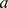 , 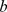 , 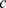 , 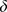 , 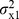 , 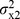 , *x = A B*, 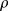
	MS4	Independence	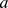 , 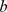 , 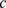 , 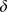 , 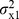 , 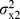 , *x = A B*, 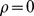
Multi-Year - including yearly, spatial and size correlation	MY1	Unconstrained year-size correlation with species specific size correlation	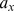 , 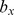 , 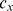 , 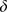 , 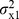 , 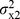 , *x = year A B*, 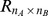
	MY2	Unconstrained year-size correlation with common size correlation	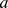 , 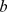 , 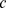 , 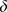 , 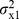 , 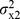 , *x = A B*, 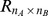
	MY3	Separable model	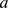 , 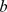 , 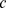 , 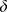 , 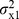 , 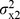 , *x = A B*, 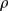
	MY4	Independence	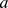 , 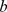 , 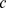 , 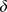 , 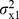 , 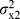 , *x = A B*, 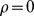

As 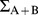 is a parameterised sub-model of the unconstrained correlation 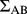 in Eq. (10), the separable model is a sub-model of unconstrained species-size correlation with common size correlation. The model states that to measure the correlation between two species A and B of sizes 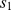 and 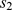, we should first transform their sizes to a common scale at which a generic covariance function 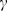 applies and finally multiply by the overall species correlation. The natural and the log scale are applied as size scaling functions. We chose the log model instead of the logistic model because they perform equally well, but the log model has fewer parameters and thus is more convenient to apply. Finally, we contrasted the model MS3, including interspecific spatial correlations, with a sub-model, MS4, for which there is no assumed species correlation (i.e., 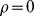) *denoted independence*.

Although multispecies models combine species, spatial and size correlations separately for each year, the exact same type of models are considered, where species and year switch roles. These models are called multi-year models, where for each species, the correlation between year, space and size is modelled.

#### 4.3 Model overview

An overview of the models considered and tested is given in Table 1, where single, multispecies and multiyear models are covered. In addition, the hierarchical structure of the model testing is indicated.

The hierarchical order of the models is as follows: 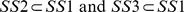


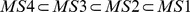


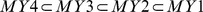



This states that the 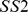 and 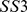 models are sub-models of the 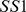 model.

Finally, we illustrate the potential of an extended correlation structure by predicting the abundance distribution of a target species, using only indirect data, i.e., data of the other species at the same year *or* the same species the year before. For the cases of cod and whiting in 2009 and 2010, the possible correlation across species and year are further investigated. We illustrate the potential of the correlation model to make spatial abundance predictions, first using species correlation models and secondly using time correlation models.

### 5. Ethics Statement

No humans, primates or laboratory animals were involved in the study. There was no sampling from private land, and the field studies did not involve endangered or protected species. Only fish sampled in public sea areas have been used. All fish were sampled with research survey trawls under or related to ICES (International Council for Exploration of the Sea; www.ices.dk) coordinated international standard trawl surveying. The sampling and handling of fish strictly followed all ICES guidelines, procedures, legislative rules, and permissions from national governments for sampling and handling of fish in fisheries research surveys. The sampling was conducted by national government-owned research vessels following Danish national legislation, permissions, and ethics for handling of wild caught fish. The sampling was performed under repeated international standardised surveying where the research vessels had full permission to sample from all relevant national public authorities (governments) in the Baltic waters.

There was no approval of this study by an Institutional Animal Care and Use Committee (IACUCO) or Ethics Committee. This was not necessary because the sampling and handling of fish strictly followed all ICES guidelines, procedures, legislative rules, and permissions from national governments for sampling and handling of fish in fisheries research surveys. The sampling was conducted by national government-owned research vessels following Danish national legislation, permissions, and ethics for handling of wild-caught fish.

## Results

### 1. Single-species models

**Figure 3:**
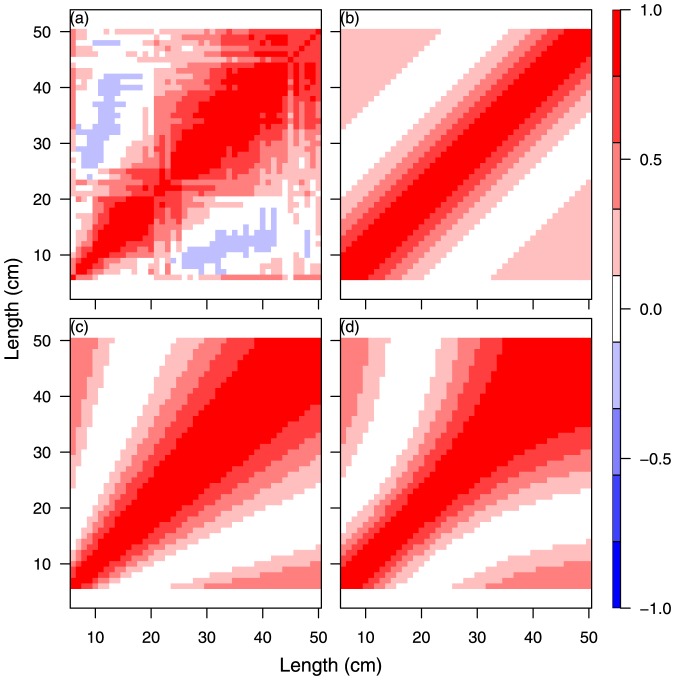
Model comparison for cod year 2010 quarter 4. SS1, unconstrained (a); SS2, natural scale (b); SS3, log scaled (c); SS4, logistic scaled (d).

The results of the four separate single species analyses, SS2-SS5, of cod and whiting in the fourth quarter in 2009 and 2010 are shown in Figures 2, 3, S2, and S3 and Table 2 and Table S1 in File S1. The different size correlation structures tested are shown separately in the 4 panels of each figure, covering an hierarchical testing procedure (see Fig. 2 text). The detailed distribution patterns of cod and whiting according to size group are described in Supporting Information Appendix A for the period 2009–2012, quarters 1 and 4.

**Table 2:** Model comparisons with unconstrained size correlation versus natural, log and logistic scaled and model reductions according to either species or year.

Model or Structure of correlation	Tot Df	Deviance	Chisq	Df	Pr(>Chisq)
Model comparison, cod year 2009 quarter 4					
(SS1) Unconstrained	993	−122858.25			
(SS2) Natural	6	−122607.85	250.40	987	1.000
(SS3) Log	6	−122700.71	157.54	987	1.000
(SS4) Logistic	8	−122693.00	165.25	985	1.000
Model comparison, cod year 2010 quarter 4					
(SS1) Unconstrained	993	−133760.09			
(SS2) Natural	6	−133241.87	518.22	987	1.000
(SS3) Log	6	−133336.41	423.68	987	1.000
(SS4) Logistic	8	−133325.86	434.23	985	1.000
Model reduction: cod+whiting year 2009 quarter 4 by structure of species correlation					
(MS1) Unconstrained species correlation	1451	−226997.63			
(MS2) Common correlation function	1449	−226984.52	13.11	2	<0.010
(MS3) Separable	10	−226433.58	550.94	1439	1.000
(MS4) Independence	9	−226430.51	3.08	1	0.079
Model reduction: cod year 2009+2010 quarter 4 by structure of year correlation					
(MY1) Unconstrained year correlation	2036	−256427.46			
(MY2) Common correlation function	2034	−256423.99	3.47	2	0.176
(MY3) Separable	10	−256055.89	368.10	2024	1.000
(MY4) Independence	9	−256028.29	27.59	1	0.000

The main purpose of the tests was to investigate for each species and year which of the size correlations models that could be rejected or not (Hypothesis H01 and the models SS2, SS3 or SS4). It was especially important to establish whether the free unconstrained model could be reduced to a structured model so that abundance predictions could be made using a model with few parameters. All models were parameterised from the same correlation function (Eq. 5) on the transformed scale. The spatial correlation parameter was first tested for independence of size and time for each species and both years, and it was found that it was possible to distinguish between the space and size-time correlation. The Chi-Square likelihood ratio tests of the different models SS1. SS2, SS3, and SS4 (Table 2 and Table S1 in File S1) did not have sufficient power to reject any of the correlation structures for both cod and whiting in 2009 and 2010 because of the very high number of degrees of freedom in the main model. This indicates that there is no significant difference between (SS1) and (SS2, SS3, and SS4) with respect to the description of the correlation between size groups for cod any of the years (Table 2), and consequently we cannot reject a structured correlation model (hypothesis H01).

Higher power of the tests can be obtained by re-binning the data to 2-cm size groups and re-fitting the models (SS1)–(SS4), as this will very much reduce the degrees of freedom of model (SS1). When using 2-cm size groups, the test results for cod in both 2009 and 2010 (not shown) came out in favour of the log- and logistic transformations parameterising a structured size correlation model, as the identity transform was rejected (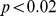). Thus, the identity transformation was excluded from the analysis. There is a trade-off between precision in the model by using 2-cm size groups compared with the high resolution in the rate of change in distribution when using 1-cm groups. In the present approach, we use the 1-cm groups to retain as much information as possible in the distribution dynamics of the fish. On average, a juvenile Baltic cod grows 1 cm in 6 weeks, and when using 2-cm size groups, the time resolution of 1.5 months is considered too high.

Consequently, a size-structured model cannot be rejected for any of the species for both years. In the multi-species and -year model extensions, the log transformation parameterisation was chosen because it is simpler than the logistic transformation in the sense that it does not contain any further parameters.

**Figure 4:**
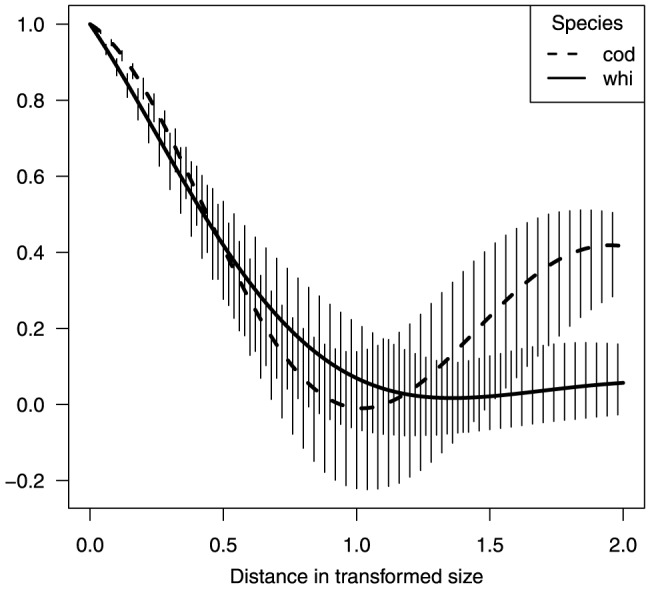
Estimated correlation (y-axis) for different size correlation functions, Eq. (5), of cod and whiting (single species runs) using the log transform model parameters given by Table 3. The vertical bars indicate the 95% confidence intervals.

An alternative criterion for model selection is parameter consistency over time. In this case, the question regarding the four independent analysis (Figs. 2, 3, S2, S3) is which one of the transformation functions for the structured size correlation models (SS2)-(SS3) will have the most robust parameter estimates. In other words, are the images Figure 2c and Figure 3c (or 2d or 3d) for cod significantly different? Likewise, is this the case for Figure S2c and Figure S3c (or S2d and S3d) for whiting? Parameter estimates related to the logistic (d) and log (c) transforms display equal consistency over time, and here we have reported the results of the log-transform (c) (Table 3). All correlation parameters (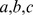) (Eq. 1) related to size can be tested independent of the year effect, and, furthermore, the spatial correlation parameter 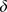 appears independent of both year and species (Table 3). It is remarkable that the parameter 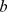
describing the angular velocity of the oscillating part (Eq. 5) of the size-correlation is significantly different for cod and whiting, explaining the main differences in the species specific size correlation functions (Fig. 4). This result basically indicates that small and large cod occur in the same spatial regions, whereas this is not the case for whiting. This pattern is also visible from the initial analyses with animations of cod and whiting abundance patterns across size-groups from the surveys as described in Supporting Information Appendix A, i.e., where the whiting is observed more westerly as smaller individuals and more easterly as larger fish, whereas small cod are observed more easterly both for the very small and very large size classes. Consequently, there is a rather consistent structure in the size correlation within the species over time.

**Table 3:** Summary of single species runs: Parameter estimates of log-transform model and size-correlation (first 5 parameters) plus spatial covariance parameters (final 9).

	Estimate	Std. Error
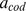	2.42	0.26
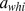	0.98	0.11
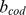	1.19	0.16
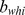	0.35	0.09
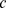	0.98	0.26
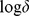	−7.02	0.57
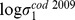	0.61	0.06
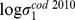	0.37	0.08
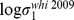	0.53	0.08
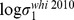	0.24	0.12
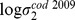	−0.47	0.14
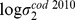	−0.05	0.11
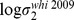	0.31	0.09
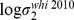	0.29	0.09

Valid parameter reduction applied over time and for some species parameters.

### 2. Multispecies models including species correlation

The next step is to use the model to assess the possible correlation between species to test hypothesis H02. This is accomplished by comparing the four multispecies correlation models MS1–MS4 and testing whether the free, unconstrained species-size model MS1 can be reduced to the sub-models M2–MS4.

**Figure 5:**
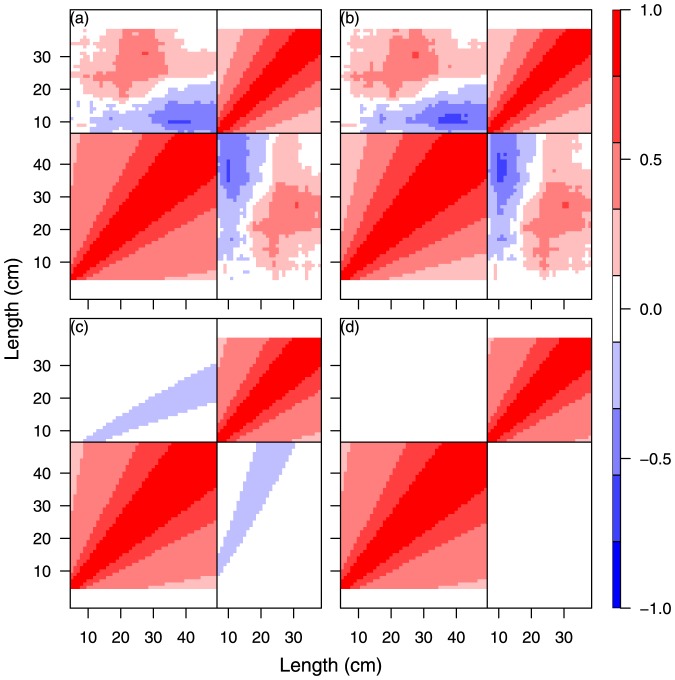
Multispecies models for cod and whiting year 2009 quarter 4. MS1, unconstrained species correlation with separate parametric size correlation for each species (a); MS2, unconstrained species correlation with common parametric size correlation for both species (b); MS3, separable species-size correlation (c); MS4, no species correlation (d).

The combined analyses with the multi-species extension of the model considering species correlation involved runs under the log-transformed size for both species (Figs. 5, 2009 and S4, 2010). The results are presented in Figure 5 for 2009 and Figure S4 for 2010 and are covered in detail in the panel (a) that presents an image of the combined correlation of cod (the large square block) and whiting (the smaller square block) using the previous (separate) analysis for each species combined with the assumption of unconstrained species-size correlation between the two species. The next panel (b) is visually very similar to panel (a) and represents the model reduction where cod and whiting are assumed to have a common correlation function. Despite the visual similarity between panel (a) and (b), the likelihood ratio test strongly rejects this reduction (Table 2). Panel (c) is an image of the separable model reduction, and this model does not fit very well. Finally, panel (d) shows the model fit, assuming species independence. The likelihood ratio tests for (c) and (d) indicate that it is not possible to distinguish between (b), (c) and (d). In summary, none of the model reductions are valid because of the rejection of model (b) because of the combined nested structure of the test procedure. Consequently, (a) will have to be chosen as final model in this case, i.e., no constraints in the species correlation. The conclu are the same for cod and whiting in 2010 (Fig. S4), except that here the separable model visually appears to perform better than for 2009, and the species correlation is significantly non-zero. Below, we return to the overall conclusions on species correlation.

### 3. Multiyear analyses including year correlation

**Figure 6:**
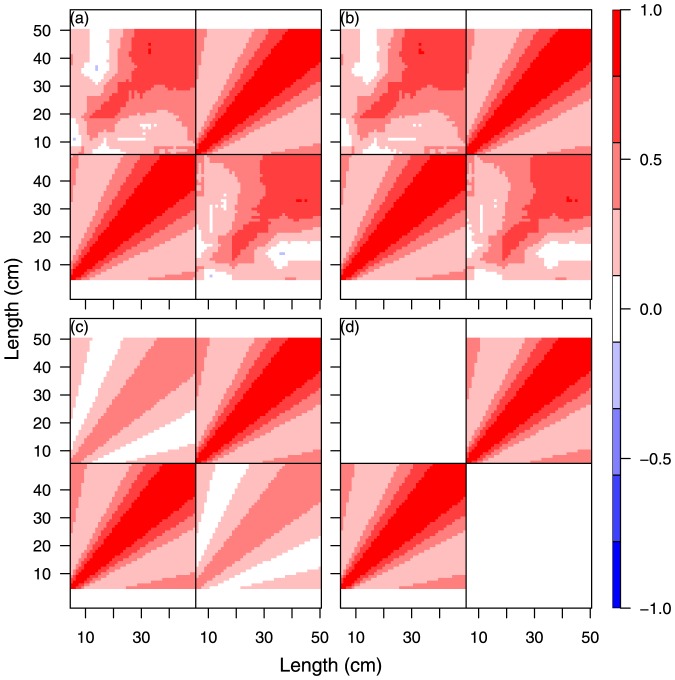
Multiyear models for cod year 2009 and 2010 quarter 4. Unconstrained year correlation with separate parametric size correlation for each year (MY1, a), Unconstrained year correlation with common parametric size correlation for both years (MY2, b), separable year-size correlation (MY3, c), No year correlation (independence) (MY4, c).

In MY1–MY4, the cod-whiting species correlation has been replaced by the corresponding 2009–2010 year correlation to test hypothesis H03. For both species (cod 2009 and 2010 in Fig. 6, and whiting 2009 and 2010 in Fig. S5), the common correlation function hypothesis in (b) cannot be rejected as 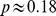 and 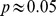, for cod and whiting, respectively (Table 2 and Table S1 in File S1). Furthermore, the separable model (c) cannot be rejected, but the independence test (d) is rejected for both species. Consequently, for both species, one will select model (c), i.e., a separable correlation model, as the final model.

### 4. Summary of the results

Three main conclusions can be drawn. i) For the single species models, the size correlation models parameterised on natural (SS2), log (SS3) and logistic scale (SS4) cannot be rejected compared with the unconstrained model (SS1). ii) The unconstrained species and year correlation models MS and MY 1–3 are all generalizations of the single species models SS 1–4. iii) Both multispecies and multiyear correlations (unconstrained) appear to occur (see Figures 5, 6, S4, S5 panels (a) and (b)). For the multiyear correlation models, it was possible to obtain a parameterised year correlation model, the separable model MY3, which was not rejected, and this indicates that Hypothesis H03 can be accepted. In contrast, we cannot reject that species correlation occurs (hypothesis H02), but this correlation is very complex and resisted the parameterisation described here. The current models appear to perform better when describing time correlation than species correlation. A free, unconstrained species-size correlation model with many parameters is therefore still needed for describing the correlation between species.

### 5. Predicting abundance surfaces

**Figure 7:**
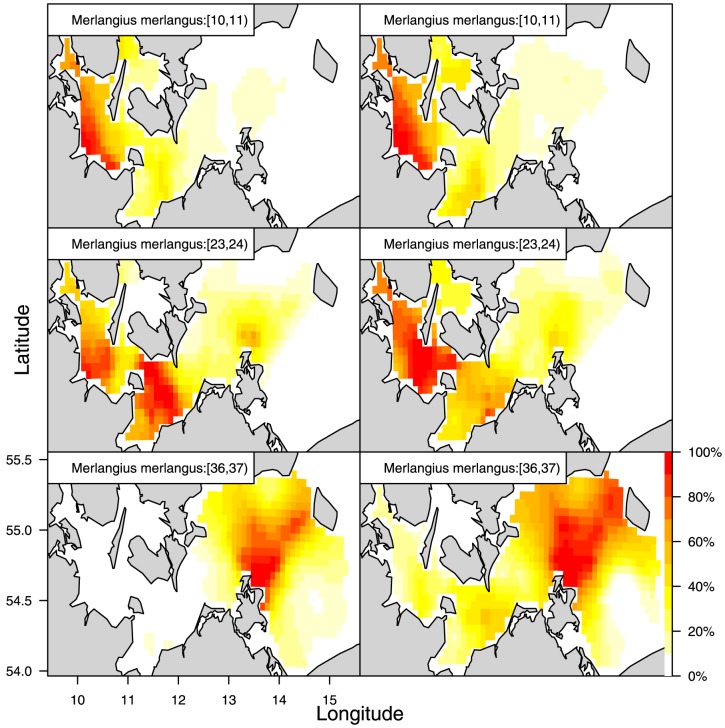
Maps of relative whiting abundance 2009/Q4 *based on whiting observations* (left column) versus the same maps *based on cod observations* (right column) utilizing MS1 model of Table 2. The three row panels indicate three whiting size groups in cm.

**Figure 8:**
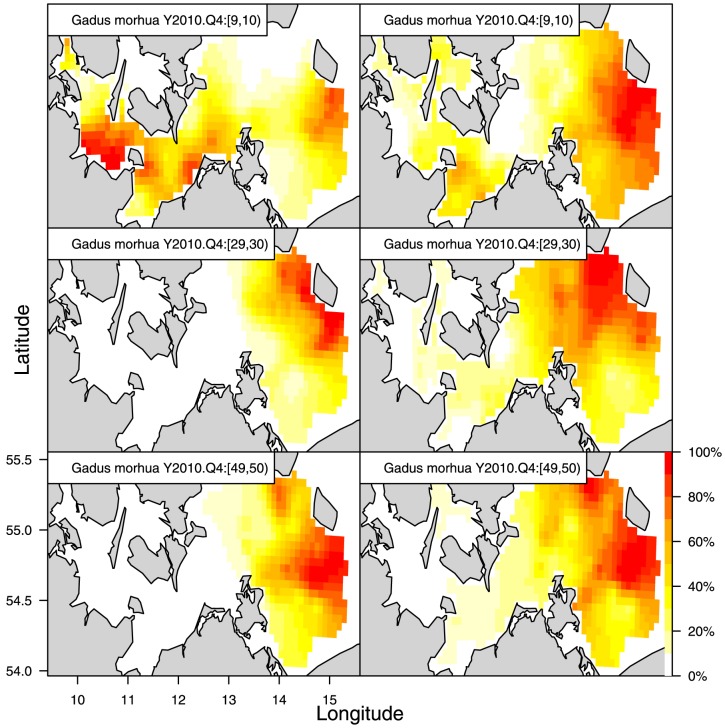
Predictive power of the models illustrated by maps of relative cod abundance 2010/Quarter 4 based on *cod observations that year* (left column) versus the same maps based on *cod observations previous year* (right column) utilizing model MY3. The three row panels indicate three cod size groups in cm.

The potential of the correlation model to make abundance surface predictions was analysed, first using (unconstrained) species correlation models (Figs. 7 and S6) and second using time correlation models (Figs. 8 and S7). An extended species or time correlation structure is useful to predict abundance surfaces of a target species when using only indirect data, i.e., data of the other species at the same year *or* the same species the year before. For all figures, the left panels represent the “observed” patterns (single species model predictions), and the right panels represent the corresponding predicted panels. The visual inspection reveals that of the performance of all predictions, the species-based predictions are perhaps the most accurate. These findings also support that we cannot reject hypothesis H02 when using a much more complex model in this comparison, i.e., the unconstrained species correlation model with many parameters, to perform the species-based predictions.

## Discussion

### 1. Trawl survey analysis model development and general application

A length-based stochastic model of single-species stock dynamics including densities [6] was applied to the Baltic cod species based exclusively on survey data; however, this model was not spatially explicit. In the present study, an extension of the statistical LGCP model [3] is applied to the standard and extended BITS data for Baltic cod and whiting to investigate not only intraspecific size correlations, including spatial and temporal distribution patterns, but also potential interspecific correlation between species in relative density according to space, size, and time.

The motivation for developing size-based density models including species, time and spatial correlation is based on the apparent visual relationship between species from sequential abundance maps (as for instance presented in Figures 7, 8, S6, S7, first column, illustrating the spatial distribution by species by size group as well as described in Supporting Information Appendix A). Quantification and modelling of the covariance functions is performed either for the same species at different time periods or for different species at the same time. The aim is to empower spatial predictions of relative density of fish within and across species after constructing spatial abundance models that support hypotheses testing regarding alternative model specifications. Such species/size time/size correlation models are high dimensional, and model reduction is sought to apply the models for predictions. We formulated natural model reduction hypotheses based on a size transformation that results in fish being able to be compared on a size scale.

### 2. Structure of size correlation models by species and size transformations used for model parameterisation

The separate single species analyses assumed that the spatial and the size correlations in density are independent among species. The analyses further revealed that the idea that fish can be compared on a size scale by transformations of the natural size scale cannot be rejected for any of the species for both years, and there is a rather consistent structure in the size correlation within species over time. The log and logistic size transformations, but not the natural (no transformation) for cod, were not rejected when changing the bins to 2-cm length groups. On this basis, hypothesis H01 cannot be rejected. As such, the model enables prediction, interpolation and animation of unobserved relative distribution and density patterns at any location and season of the year in the area for, e.g., cod and whiting.

### 3. Multi-species and multi-year correlation models according to model complexity

Regarding model complexity, the following conclusions have been drawn. For the relation between a year and the subsequent year of the spatial distribution of a given species/size, the simple reduced low dimensional model is adequate to describe the complicated observed correlation patterns, and a significant correlation between years was found. For the multispecies relationship, the simple structured models developed are not adequate to describe the correlation pattern, and thus, we were not able to decide if a significant species correlation exists. This indicates that the separable model is found too simple to describe the potential species/size correlation. However, it is remarkable how well the (unconstrained) species correlations model can predict a “missing” species, which indicates that species correlation may exist, and we cannot reject hypothesis H02. In general, the strength in the approach lies in the detailed description and testing of the combination of species-size and time-size correlations.

For the single species models, area and time spatial variations in log fish density, 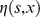, of a point in the space, 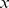, for a given size are assumed random by nature. However, if some structure in relative fish densities according to animal behaviour exists, we expect to observe fish of similar sizes occupying the same spatial areas. For the two competing species, cod and whiting, we expect that fish of similar sizes of occupy the same spatial areas (sharing the same habitats, food sources, etc.) or fish of different sizes occupy the same spatial areas (due to predation on each other and even potential cannibalism). Both cod and whiting have, for the North Sea, been demonstrated to be competing species for the same habitats and to predate on each other (e.g., [13]). Interspecific relationships may play a role in the distribution patterns of WBC cod and whiting, but this phenomenon is not well understood [27], [28], [29], [19]. There is spatial and temporal variation in biological interactions due to predation by cod in the Baltic Sea, where also cannibalism has been documented as an impacting factor in certain periods [30], [31], [20], [12], [32]. The levels of cannibalism are dependent on the abundance of juveniles and larger cod predators, their overlap in distribution, and the availability of alternative prey items for larger cod, such as sprat and herring [33], [34], [20], [32]. In the WBS, there are also abundant competing gadoid predators in the form of whiting [12].

The basic single species model used (Eq. 1) includes three processes: large spatial scale variations for each size group, small-scale variations and a spatial/size correlation component describing the spatial landscape by size. The large-scale variation is chosen as generally as possible, i.e., an unconstrained model including a parameter for each size group. The small-scale variation and correlation are assumed to be the same for each point in the space and depends only on the size correlation. The spatial/size correlation was modelled using a Gaussian random field, for which all possible ways between two spatial points are evaluated and for which shorter paths are preferred to longer paths. This model has the advantage that paths crossing land can be avoided.

For the multispecies models including species correlation, it was assumed that the parameters affecting the degree of spatial correlation are assumed to be the same for the species considered. This may be incorrect for species with different spatial behaviour. Similarly, for the multiyear analyses, it was assumed that these parameters are the same for the time periods considered.

Whether all of these assumptions are too restrictive or not depends on the species and time periods considered and must be tested statistically in each case. The results indicate all single species models and assumptions for cod and whiting in the WBS are not rejected. The same applies to the multiyear analyses, whereas the multispecies structured models are rejected.

It should be noted that estimation of spatially aggregated absolute abundance demands correction for bias in the log-normal distribution, especially if data are far from Gaussian. However, for constant variance fields, the correction has no effect on relative abundance as used here. Estimation of absolute abundance is usually performed by posterior simulation as demonstrated by, e.g., Lewy and Kristensen [2]. The present paper does not include this problem because we consider all mean value parameters, 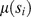, as nuisance parameters where we operate with relative abundance surfaces rather than simulating and predicting absolute abundance.

### 4. Further extension of the correlation structures and future studies

In the present analyses, we assume that there is no difference in the small- and large-scale size correlations [3]. In addition, we assume that the small-scale variations, 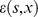, i.e., the within haul variations, are correlated across size-classes because of possible size-dependent schooling [3]. As small-scale variations can potentially be dominating, future studies should analyse differences in the large scale and small scale variation in relation to species, and an improved model should take into account differences between large-scale and small-scale variation.

For the multi-species extension, a structured size correlation model is used where correlations between size classes only depend on the distance between transformed sizes and where a common parameterisation on the different size transformation scales are applied (involving log transformed size) for both species. The same function for distance between transformed sizes does not necessarily need to be used for both species. Figure 4 indicates that the parameter 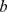, which describes the angular velocity of the oscillating part of the size-correlation, is significantly different for cod and whiting, explaining the main differences in the species specific size correlation functions when applying the same size correlation structure (i.e., the same size class transformation). Higher correlation between species might have been achieved by applying different functions according to distance in transformed size where more complex species specific structures are taken into account. Future studies should investigate further alternative species specific functions for transformation of size to parameterise the same size correlation model.

The present analyses have only covered model development and data analysis of observations from relatively few years, quarters and species. Further studies should investigate the structure of the correlation models and their size transformation parameterisations as well as the multi-species correlation models for an extended set of years, quarters and fish species.

The purpose of our study was to establish a correlation structure describing the spatial distribution and relative density patterns of a single species of all size-classes *for a time-snapshot* (1 month). Our modelling of animal density is based on considerations and testing of the processes acting on a spatial scale using survey catch rates by size group by haul. Alternatively, future studies could consider combining the existing area-based time snapshot models with new models tracking the movements in time. Perhaps such models modelling the correlation between the directional movements could better capture the fish behaviour and the resulting spatial fish distribution.

Finally, model-based geostatistical methods can be further applied to investigate optimal survey designs for different species and size groups using the extended BITS survey dataset established here.

## Supporting Information

Figure S1 **Spatial correlation measured from given centre point.** It shows the properties of the Gaussian Markov Random field co-variance (Q^−1^) generated from Eq. 4, which indicates a decreasing correlation according to distance taking into to account the geometry of the grid. The co-variance (correlation) depends on all possible ways between two points, i.e., it is an integral over all possible ways between the centre point and any other point weighted with the distance of the way (in the sea and not over land).
(TIF)

Figure S2 **Model comparison for whiting year 2009 quarter 4.** SS1, unconstrained (a); SS2, natural scale (c); SS3, log scale (d); SS4, logistic scaled (d).
(EPS)

Figure S3 **Model comparison for whiting year 2010 quarter 4.** SS1, unconstrained (a); SS2, natural scale (b); SS3, log scaled (c); SS4, logistic scaled (d).
(EPS)

Figure S4 **Multispecies models for cod and whiting year 2010, quarter 4.** See figure explanation for Figure 5.
(EPS)

Figure S5 **Multiyear models for whiting year 2009 and 2010 quarter 4.** See figure explanation for Figure 6.
(EPS)

Figure S6 **Maps of relative whiting abundance 2010/Q4** ***based on whiting observations*** **(left column) versus the same maps** ***based on cod observations*** **(right column) utilizing MS1 model of Table S1 in File S1.** The three row panels indicate three whiting size groups in cm.
(EPS)

Figure S7 **Predictive power of the models illustrated by maps of relative whiting abundance 2010/Quarter 4 based on** ***whiting observations that year*** **(left column) versus the same maps based on** ***whiting observations previous year*** **(right column) utilizing model MY3.** The three row panels indicate three whiting size groups in cm.
(EPS)

File S1 **Table S1, (containing model comparisons with unconstrained size correlation versus natural, log and logistic scaled, and model reductions according to either species or year for whiting year 2009 quarter 4, whiting quarter 4 2010, cod+whiting year 2010 quarter 4, and whiting year 2009+2010).** Keywords; Appendix A (with description of specific distribution patterns for cod and whiting for different size groups); Appendix B (with description of methods on (B1) how to parameterise a general positive definite (PD) correlation matrix *φ*? R?, (B2) how to parameterise a general positive definite correlation matrix with given marginal, and (B3) the proof for this).
(DOC)
