# Correction: A Statistical Model for Estimation of Fish Density Including Correlation in Size, Space, Time and between Species from Research Survey Data

**DOI:** 10.1371/journal.pone.0148722

**Published:** 2016-02-03

**Authors:** J. Rasmus Nielsen, Kasper Kristensen, Peter Lewy, Francois Bastardie

The following information is missing from the Acknowledgements section: The research leading to these results has partly received funding from the EU-FP7-SOCIOEC project under grant agreement No. 289192.
