# Supplementary figures and images for: A Statistical Model for Estimation of Fish Density Including Correlation in Size, Space, Time and between Species from Research Survey Data
Source: PLoS One. 2014 Jun 9;9(6):e99151. doi: 10.1371/journal.pone.0099151 (PMC4049607; doi:10.1371/journal.pone.0099151)

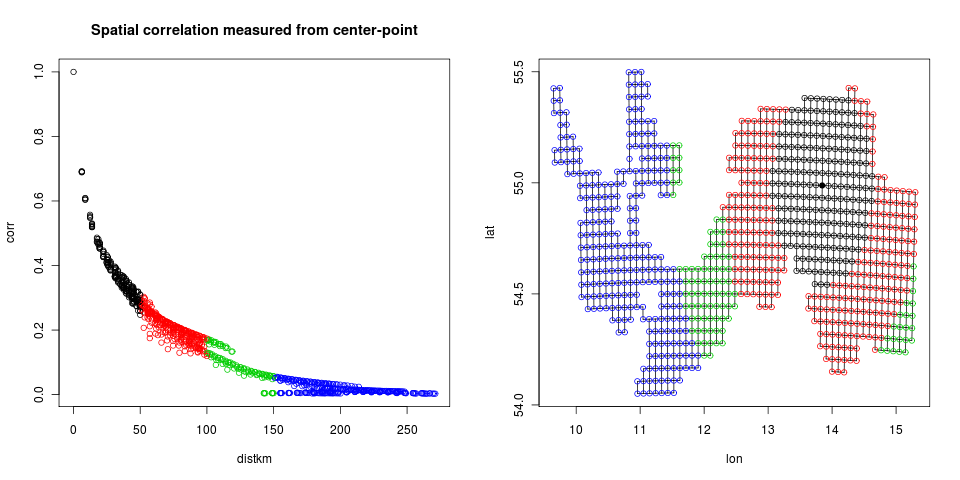

Supplement: Figure S1 — Spatial correlation measured from given centre point. It shows the properties of the Gaussian Markov Random field co-variance (Q−1) generated from Eq. 4, which indicates a decreasing correlation according to distance taking into to account the geometry of the grid. The co-variance (correlation) depends on all possible ways between two points, i.e., it is an integral over all possible ways between the centre point and any other point weighted with the distance of the way (in the sea and not over land). (TIF) [file pone.0099151.s001.tif]
