# Supplementary material for: A Statistical Model for Estimation of Fish Density Including Correlation in Size, Space, Time and between Species from Research Survey Data
Source: PLoS One. 2014 Jun 9;9(6):e99151. doi: 10.1371/journal.pone.0099151 (PMC4049607; doi:10.1371/journal.pone.0099151)
Supplement: File S1 — Table S1, (containing model comparisons with unconstrained size correlation versus natural, log and logistic scaled, and model reductions according to either species or year for whiting year 2009 quarter 4, whiting quarter 4 2010, cod+whiting year 2010 quarter 4, and whiting year 2009+2010). Keywords; Appendix A (with description of specific distribution patterns for cod and whiting for different size groups); Appendix B (with description of methods on (B1) how to parameterise a general positive definite (PD) correlation matrix φ? R?, (B2) how to parameterise a general positive definite correlation matrix with given marginal, and (B3) the proof for this). (DOC) [file pone.0099151.s008.doc]

Supporting Information

Table S1: Model comparisons with unstructured size correlation versus natural, log and logistic scaled, and model reductions according to either species or year.

| **Model or Structure of correlation** | **Tot Df** | **Deviance** | **Chisq** | **Df** | **Pr(**$>$**Chisq)** |
| --- | --- | --- | --- | --- | --- |
| Model comparison whiting year 2009 quarter 4 | | | | | |
| (SS1) Unstructured | 499 | -104258.23 |  |  |  |
| (SS2) Natural | 6 | -103730.09 | 528.13 | 493 | 0.132 |
| (SS3) Log | 6 | -103753.26 | 504.96 | 493 | 0.344 |
| (SS4) Logistic | 8 | -103741.17 | 517.06 | 491 | 0.200 |
| Whiting quarter 4 2010: Unstructured size correlation versus natural, log and logistic scaled | | | | | |
| (SS1) Unstructured | 499 | -84291.20 |  |  |  |
| (SS2) Natural | 6 | -83836.62 | 454.58 | 493 | 0.891 |
| (SS3) Log | 6 | -83849.93 | 441.27 | 493 | 0.954 |
| (SS4) Logistic | 8 | -83849.82 | 441.38 | 491 | 0.947 |
| | Model reduction: cod+whiting year 2010 quarter 4 by structure of species correlation | | | | | | | | | | | | --- | --- | --- | --- | --- | --- | --- | --- | --- | --- | --- | | (MS1) Unstructured spec. correlation | 1451 | | -217639.58 | |  | |  | |  | | | (MS2) Common correlation function | 1449 | | -217628.91 | | 10.67 | | 2 | | <0.01 | | | (MS3) Separable | 10 | | -217199.79 | | 429.12 | | 1439 | | 1.000 | | | (MS4) Independence | 9 | | -217165.00 | | 34.80 | | 1 | | 0.000 | | | Model reduction: whiting year 2009+2010 quarter by structure of year correlation | | | | | | | | | | | | (MY1) Unstructured year correlation | | 1035 | | -187928.81 | |  | |  | |  | | (MY2) Common correlation function | | 1033 | | -187922.61 | | 6.19 | | 2 | | <0.05 | | (MY3) Separable | | 10 | | -187611.44 | | 311.17 | | 1023 | | 1.000 | | (MY4) Independence | | 9 | | -187596.13 | | 15.31 | | 1 | | <0.01 | | | | | | |

Keywords

Baltic Sea; cod (*Gadus morhua*); high resolution research survey analysis; inter-specific correlation structures; log-Gaussian Cox process model; over-dispersion; species and size correlations; statistical modeling; time and space correlations; whiting (*Merlangius merlan­gus*); zero-observations.

Appendix A

This appendix gives a summary description of many distribution maps with direct observations of survey distributions by size and species. Together they indicate or “animates” movements of each species (cod and whiting) by 2-cm size groups and compare their mutual distribution patterns according to size.

With respect to the cod (*Gadus morhua*) distribution patterns in the WBS the smallest cod (1-2 cm up to 4-5 cm) are only observed in quarter 1 in 2012 with main concentrations in the south-eastern part of the WBS, i.e the Mecklenburger Bay (SD 22), and lesser concentrations of the smallest sizes in the Arkona Basin (SD 24) and the western areas of the Bornholm Basin (SD 25). As 3-5 cm they become more concentrated in the two latter areas. As 5-6 cm they are observed in both quarters of 2011 and in quarter 4 2009 besides quarter 1 2012 also with main concentrations in the two latter areas. From 6-7 cm juvenile cod are observed in all years and both quarters, and for the 6-8 cm cod the main concentrations are consistent in the Arkona Basin and in the western Bornholm Basin both in quarters 1 4 with different smaller concentrations also more westerly located up to the Belt Sea (SD 22). From 8-12 cm there is a tendency towards a westward shift in concentrations so main concentrations are both in the Arkona and Bornholm Basins and in the different more westerly areas including the Belt Sea. From 17-18 cm in quarter 4 and from 20-30 cm (depending of year) in quarter 1 the cod again gradually concentrate in the Arkona Basin and in the western Bornholm Basin. This continued higher concentration in those areas is consistent for all years and both quarters, however, in quarter 1 there are as well concentrations in the Mecklenburger Bay and the Belt Sea of large cod from size 40-48 cm and bigger (start size depend on year), while in quarter 4 the biggest cod larger than 54 cm are not observed in any year in the whole area. In general the yearly and seasonal (q1 and q4) main concentration areas of cod are similar between length groups. In general the bigger cods of sizes above 20 cm concentrate more in very distinct areas compared to the smaller cod, while the smaller cod are more widely distributed but still with different areas of concentrations as described above.

With respect to whiting (*Merlangius merlangus*) distribution patterns, then the small whiting from 6-7 cm up to around 12-13 cm show concentrations in the most south-westerly parts of the WBS including the Belt Sea both in quarter 1 and quarter 4, i.e. they are present in both quarters all years. From 13-14 cm up to around 35 cm in size the whiting show a gradually increasing eastward concentration as well in the Arkona Basin both in quarter 1 and quarter 4 with a shift towards highest concentrations in the Arkona Basin area from size 30 cm. The biggest whiting from 35 cm disappear from the western parts of the WBC and only concentrate in the Arkona Basin in both quarters 1 and 4, and the very largest whiting from around 39-40 cm and bigger are not observed in the WBC in quarter 4. In general, the smallest juvenile whiting show very westerly concentrations and are not as widely distributed as the juvenile cod both in q1 and q4. Bigger whiting both show concentrations in the westerly areas as well as in the easterly Arkona Basin of the WBC, and the largest whiting show only concentrations in the Arkona Basin both in q1 and q4.

In comparison, the cod and whiting resembles each other in relation to their main concentrations are located in the same areas in both quarters 1 4, however, the smaller whiting do not concentrate in the eastward areas of the WBS such as the Arkona Basin and the western Bornholm Basin as the juvenile cod also do with their wider distribution in general compared to the whiting. For the bigger whiting from size 30 and cod from size 30 and bigger there is a common concentration in the Arkona Basin in both quarters, but cod are also concentrating in the western Bornholm Basin and in the Mecklenburger Bay which is not observed for whiting. Accordingly, alignment in the model for cod and whiting seems to be best for the 30-40 cm groups followed by 20-30 cm groups of both species in both quarters.

Appendix B

### *B.1 How to parameterize a general positive definite (PD) correlation matrix* *? R?*

For simplicity consider the case $n=3$. Define

$$L=\left(\begin{matrix} 1 & & \\ L_{21} & 1 & \\ L_{31} & L_{32} & 1 \\ & & \end{matrix}\right)$$

The diagonal of $LL\text{'}$ is equal to the squared row-sums of $L$, knowing *L´* the transpose of matrix *L*. Define the diagonal matrix $D=diag\left(LL\text{'}\right)$:

$$D=\left(\begin{matrix} 1 & & \\ & 1+L_{21}^{2} & \\ & & 1+L_{31}^{2}+L_{32}^{2} \\ & & \end{matrix}\right)$$

Then the matrix

$$D^{-\frac{1}{2}}\left(LL\text{'}\right)D^{-\frac{1}{2}}$$

defines a positive definite matrix with variance one in each coordinate direction (a correlation matrix). The above description suggests the mapping (parameterization):

Can a proof be found to demonstate that we hit all positive definite 3 by 3 correlation matrices?

###

### *B.2 How to parameterize a general positive definite correlation matrix with given marginals*

First consider the case of identity marginals. Define

$$L=\left(\begin{matrix} I_{A} & \\ R & I_{B} \end{matrix}\right)$$

$$LL\text{'}=\left(\begin{matrix} I_{A} & R\text{'} \\ R & I_{B}+RR\text{'} \end{matrix}\right)$$

$$D=\left(\begin{matrix} I_{A} & \\ & I_{B}+RR\text{'} \end{matrix}\right)$$

We guess that

$$D^{-\frac{1}{2}}\left(LL\text{'}\right)D^{-\frac{1}{2}}$$

will hit all the desired matrices (as function of the rectangular matrix $R$). The general case with marginals $\text{Σ}_{A}$ and $\text{Σ}_{B}$ is then found by scaling with

$$\left(\begin{matrix} \text{Σ}_{A} & \\ & \text{Σ}_{B} \end{matrix}\right)^{\frac{1}{2}}$$

###

### *B.3 Proof*

We have to show that the mapping

is a bijection, where $L_{R}$ and $D_{R}$ are defined by

$$L_{R}=\left(\begin{matrix} I_{A} & \\ R & I_{B} \end{matrix}\right)\text{ }D_{R}=\left(\begin{matrix} I_{A} & \\ & I_{B}+RR\text{'} \end{matrix}\right)$$

Writing out the definition of we have

First thing to notice is that is well-defined. This is because $I_{B}+RR\text{'}$ has eigenvalues in the range $\left[1,\infty\right)$ so the inverse square root $(I_{B}+RR\text{'})^{-\frac{1}{2}}$ exists.

It is directly clear that is positive definite.

Finally we have to verify that is actually able to hit any matrix in the range-space. So, for any given positive definite matrix of the form

$$\text{Σ}=\left(\begin{matrix} I_{AA} & \text{Σ}_{AB} \\ \text{Σ}_{BA} & I_{BB} \end{matrix}\right)$$

we show the existence of an such that .

The fact that $\text{Σ}$ is positive definite ensures that the Schur-complement

$$I_{B}-\text{Σ}_{BA}\text{Σ}_{AB}$$

is positive definite. It is therefore possible to construct the $n_{B}\times n_{A}$ matrix

$$R=(I_{BB}-\text{Σ}_{BA}\text{Σ}_{AB})^{-\frac{1}{2}}\text{Σ}_{BA}$$

Note, that the matrix $(I-X)^{-\frac{1}{2}}$ will generally commute with $X$ giving the simplification rule

$$RR\text{'}=(I_{BB}-\text{Σ}_{BA}\text{Σ}_{AB})^{-1}\text{Σ}_{BA}\text{Σ}_{AB}$$

Now verify the lower left corner of :

$$(I_{B}+RR')^{-½}R$$

$$=(I_{B}+(I_{B}-\sum_{BA}\sum_{AB})^{-1}\sum_{BA}\sum_{AB})^{-½}(I_{B}-\sum_{BA}\sum_{AB})^{-½\sum_{BA}}$$

$$=((I_{B}-\sum_{BA}\sum_{AB})+\sum_{BA}\sum_{AB})^{-½\sum_{BA}}$$

and conclude that .
